# Mr. Wadd, on Cold Water in the Gout

**Published:** 1803-03-01

**Authors:** S. Wadd

**Affiliations:** Basinghall Street, London


### *To the Editors of the Medical and Physical Journal.*

GENTLEMEN,

NOT being a constant reader of your Publication, I should not have seen Dr. Kinglake's account of what he calls "his new mode of treating the Gout," but for a medical friend who shewed me the last Number (No. 48) within these two days. I am greatly gratified that the subject is brought forward, and particularly so on account of the success the Doctor has had in persuading his arthritic patients to follow his advice.

It is well known, in a large circle of my acquaintance, that I have made use of cold water, internally and externally, for the gout for nearly twenty years. Externally by wet cloths, and immerging the limb in the approach and every stage of the fit. I have even exhibited before medical men of the first eminence, and gentlemen of sense, not of the profession, but subject to the disease. By the first I have been pronounced RASH, and by the last called BOLD; but, unfortunately, cannot boast of a single convert. Whenever opportunity offered I have argued the rationality of the practice, it being a favourite topic, and maintained it on the theory I had imbibed of the disease, and which I have been strongly urged to put into print, but have been deterred lest the words rash and bold should be changed to MAD.

Thank God, I have not paid the debt so long predicted by my brethren of the faculty; and I pray for long life, for, should my death happen short of ninety, it will be attributed to the use of cold water in the Gout, and may prevent me Christian burial,—so strong is prejudice.

I beg

I beg, Gentlemen, you will accept an apology for not being more acquainted with your useful work. I have not yet seen the beginning of this subject in No. 33, but I shall not lose sight of that and what may appear hereafter. I was willing to be thus early in the field to countenance the good work, and may come forth again when I can corroborate my own opinion by that of others. I am now creeping out of the severest fit I ever had; I plunged the limb into cold water at the height of the inflammation, when pain was excessive, and not to be subdued by opium. I obtained temporary ease.

I am, &c.

*Basinghall Street, London,*
*Feb.* 14, 1803.

S. WADD.

